# Case report: Fulminant type 1 diabetes following paucisymptomatic SARS-CoV-2 infection during late pregnancy

**DOI:** 10.3389/fendo.2023.1168927

**Published:** 2023-04-04

**Authors:** Lingling Zhou, Huanjia Qu, Qiuling Zhang, Jinhua Hu, Lan Shou

**Affiliations:** Department of Endocrinology and Metabolic Disease, Affiliated Hospital of Hangzhou Normal University, Hangzhou Normal University, Hangzhou, China

**Keywords:** fulminant type 1 diabetes, coronavirus disease 2019 (COVID- 19), severe acute respiratory syndrome coronavirus 2, pregnancy, case report

## Abstract

**Background:**

Dysregulation of glucose metabolism has been linked to SARS-CoV-2 infection. In addition, the occurrence of new onset diabetes mellitus, including fulminant type 1 diabetes, has been reported after SARS-CoV-2 infection or vaccination.

**Methods and results:**

A young Chinese woman in her last trimester of pregnancy presented with an abrupt progression of hyperglycemia and ketoacidosis, but with a near-normal glycohemoglobin level following paucisymptomatic SARS-CoV-2 infection. The low C peptide levels, both fasting and postprandial, reflected profound insulin deficiency in the setting of negative islet autoantibody testing, consistent with a diagnosis of fulminant type 1 diabetes. Ketoacidosis and hyperglycemia quickly improved following the introduction of insulin therapy, but not the β cell function. The patient received treatment with insulin pump therapy after being discharged, and the first follow-up revealed a well-controlled glucose profile.

**Conclusions:**

New-onset FT1D can occur after SARS-CoV-2 infection. Our report raises awareness of this rare but serious situation, promoting early recognition and management of FT1D during the COVID-19 pandemic.

## Highlights

1. It is unknown if the risk of long-term diabetes mellitus incidence increases, but SARS-CoV-2 infection causes aberrant glycometabolism.
2. FT1D often follows a preceding viral infection in a susceptible individual. There have been a few cases of FT1D after SARS-CoV-2 infection or vaccination.
3. SARS-CoV-2-induced islet dysfunction likely occurs not only *via* direct viral entry but also *via* inflammation and oxidative stress systematically or in islet micro-environment.
4. During the COVID-19 pandemic, attention should be paid to identify FT1D.

## Introduction

There are mixed epidemiological results on the association between new-onset diabetes and infection by severe acute respiratory syndrome coronavirus 2 (SARS-CoV-2). Studies from some regions have reported an increased type 1 diabetes mellitus (T1DM) incidence during the pandemic, which supports the diabetogenic effect of COVID-19, while other studies have not (1, 2). Fulminant type 1 diabetes (FT1D), a relatively rare subtype of T1DM first reported in Japan, is characterized by a rapid progression of insulin deficiency at disease onset (3). The diagnosis is based on the following: i) occurrence of diabetic ketosis soon after the onset of hyperglycemic symptoms, ii) high level of plasma glucose (≥16.0 mmol/L, ≥288 mg/dL) but a relatively mismatched level of glycohemoglobin (<8.7%), and iii) presence of endogenous insulin deficiency including urinary C peptide excretion <10 μg/day or fasting serum C peptide level <0.3 ng/ml (<0.10 nmol/L) and <0.5 ng/ml (<0.17 nmol/L) after intravenous glucagon or after meal load without verifiable islet-related autoantibodies (4). Though the etiology of FT1D has not been fully elucidated, viral infection is considered to be the most important environmental risk factor for FT1D (4).

Recently, several case reports of FT1D after COVID-19 vaccination or infection were mainly on patients from East Asia (5). Here, we describe a pregnant woman who eventually experienced a rapid clinical course of diabetic ketoacidosis following SARS-CoV-2 infection, drawing attention to FT1D during the COVID-19 pandemic.

## Case presentation and diagnosis assessment

A 34-year-old Chinese woman with a singleton pregnancy in her 34th gestational week presented to our hospital due to the abrupt onset of polydipsia, nausea, and vomiting for one day. Five weeks prior to admission, she had a brief fever with a maximum temperature of 37.8°C, accompanied by mild fatigue and muscle pain lasting for two days. She did not seek additional medical attention despite a positive COVID-19 antigen self-test at that time. She had been regularly evaluated in the obstetrics department for close follow-up and her OGTT test result was negative at the gestational age of 25 weeks. She was in good health with a normal weight before pregnancy (53 kg and a BMI of 21 kg/m^2^) and was not on any medication or vaccination, and did not smoke or consume alcohol during pregnancy. None of her family members had a history of type 1 or type 2 diabetes mellitus.

At admission, the patient appeared to be dehydrated but was conscious. A quick physical examination revealed a heart rate of 108 beats/minute, blood pressure of 132/72 mmHg, temperature of 36.6°C, respiratory rate of 20 breaths/minute, and oxygen saturation of 98% breathing ambient air. The patient was 159 cm in height and 67 kg in weight, with a BMI of 26.5 kg/m^2^. A physical examination of the abdomen revealed no upper abdominal pain, guarding, or rebound tenderness. The symphysis-to-fundal height was 30 cm, and the abdominal circumference was 102 cm. On obstetric evaluation, she was found to have uterine contractions every 20 s per 2 min. The fetus presented as a vertex presentation with an unengaged fetal head. Electronic fetal heart rate monitoring showed a fetal heart rate (FHR) of 114 bpm and an unsatisfactory contraction stress test (CST) result. Emergency obstetric ultrasound showed a biparietal diameter (BPD) of 88 mm, femur length (FL) of 69 mm, FHR of 110 bpm, and a single deepest vertical pocket of 70 mm.

Initial lab work showed an arterial pH of 7.08, actual bicarbonate of 8.6 mmol/L, β-hydroxybutyrate of 5.8 mmol/L, glucose level of 29 mmol/L (522 mg/dL), sodium level of 125 mmol/L, potassium level of 5.8 mmol/L, chloride level of 93 mmol/L, white cell count of 25.6×10^9^/L, hemoglobin level of 114 g/L, and mildly elevated pancreatic enzymes (amylase and lipase levels less than 3*upper limit of normal). The lipids profile indicated a severe hypertriglyceridemia level of 10.99 mmol/L. Further laboratory tests revealed a glycohemoglobin level of 5.9%, a glycated serum protein level of 1.45 mmol/L, and an extremely low serum C peptide level of 0.02 ng/ml. Immunological examination yielded an absence of serum islet autoantibodies, including glutamic acid decarboxylase antibody (GADAb), insulinoma associated antigen-2 antibody (IA-2Ab), islet cell antibody (ICAb), and insulin antibody(I-Ab). She tested positive for IgG but not IgM antibodies against SARS-CoV-2. Other potential viral antibodies, including coxsackievirus, cytomegalovirus, parainfluenza virus, human herpes virus, and Epstein–Barr virus, were also tested, but the results returned negative. We ruled out acute pancreatitis from an abdominal computed tomography scan, with no signs of pancreatic edema or exudation soon after the patient received urgent Cesarean section surgery.

## Diagnosis

She was diagnosed with diabetic ketoacidosis (DKA) and fulminant type 1 diabetes mellitus.

## Treatment

The patient underwent emergency C-section surgery for acute fetal distress and was immediately treated with fluid resuscitation, intravenous insulin infusion, and low-dose sodium bicarbonate. Her main complaints, metabolic acidosis and electrolyte disturbance, were solved within six hours. Two days after admission, β-hydroxybutyrate level was 0.15mmol/L. She was then switched to a subcutaneous insulin regimen for glycemic control and was initiated with fenofibrate to lower the triglyceride level. Her initial subcutaneous insulin regimen was insulin Glargine 14u SC QHS and insulin aspart 6u SC before meals. On the tenth day after admission the patient started to use an insulin pump and over the next few days the glucose levels stabilized at 4.5–15.6 mmol/L (81–281 mg/dL).

## Outcome and follow-up

The newborn infant had a poor Apgar score (4/10) at birth, which was re-evaluated to be 3/10 (at both 5 min and 10 min after birth), and was transferred to a neonatal unit but died afterward. After the C-section, the patient was transferred to intensive care unit and then the endocrine ward. She was hospitalized for another two weeks and then was discharged on continuous subcutaneous insulin infusion therapy. However, three weeks after the disease onset, she still had profound insulin deficiency, as evidenced by low levels of and a slight but unsatisfactory increase of C peptide in the fasting state and after a mixed meal load (shown in **Table 1**).

**Table 1:** Results of mixed meal tolerance test in the 1st follow-up and OGTT at the 25th gestational week.

	Glucose level (mmol/L)	C peptide level (ng/ml)
Mixed meal load* in the 1st follow-up		
Fasting	6.57 (118 mg/dL)	0.02
Postprandial (2 h)	21.39 (385 mg/dL)	0.05
OGTT at the 25th gestational week		
Fasting	3.64 (66 mg/dL)	Not applicable
Post oral glucose (1h)	8.67 (156 mg/dL)	Not applicable
Post oral glucose (2h)	6.66 (120 mg/dL)	Not applicable

* The meal contained approximately 70 g carbohydrates, 24 g proteins, and 15 g fat.

## Discussion

**Figure 1:**
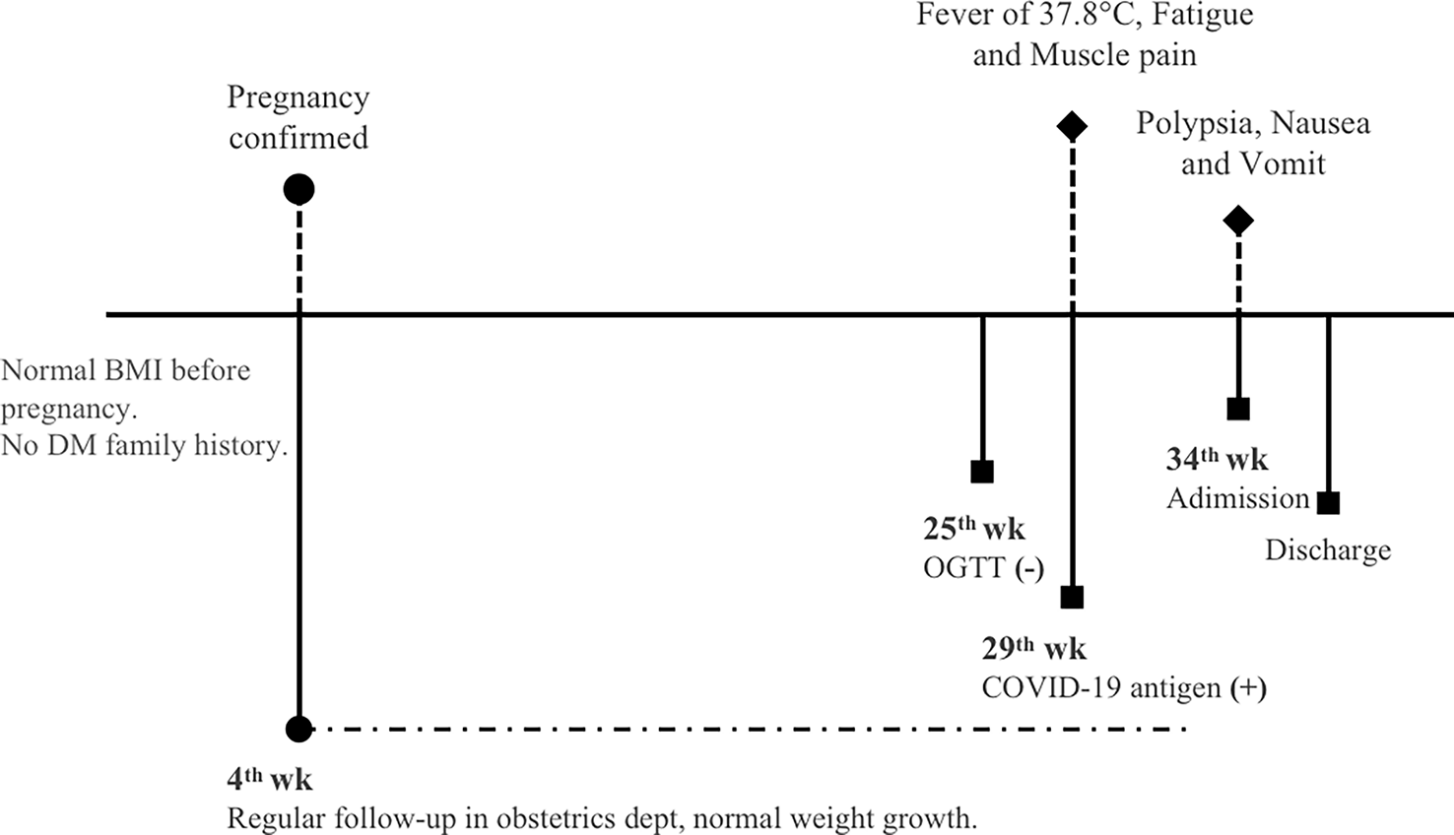
Timeline of events and important information of this patient.

To the best of our knowledge, this is the first case of newly diagnosed fulminant type 1 diabetes following SARS-CoV-2 infection during pregnancy. The patient reported herein presented with hyperglycemia, ketoacidosis with an extremely rapid course, a near-normal glycohemoglobin level, and exhaustion of endogenous insulin secretion but without evidence of islet-related autoantibodies, which fulfilled the diagnosis of FT1D (4). Although the gestational status itself is a pre-existing risk factor for FT1D, we still suspect that in this case, FT1D occurred as rare organ damage in the pancreas due to a previous COVID-19 infection, according to the timeline of the medical history (shown in **Figure 1**), since no positive results of other suspicious virus infections reported to be associated with FT1D were obtained.

During the COVID-19 pandemic and the rolling back of strict anti-COVID-19 restrictions in China, cases of autoimmune-mediated disorders, including Graves’ disease, type 1 diabetes, Guillain‐Bare Syndrome, and systemic lupus erythematosus have been reported following SARS-CoV-2 infection or vaccination. Although the underlying mechanisms and whether SARS-CoV-2 affects pancreatic islets or other endocrine organs remain unknown, SARS-CoV-2 infection does result in aberrant glycometabolic control (6). Existing clinical data suggest that the infection can aggravate insulin resistance, increase hepatic glucose production, and impair peripheral glucose uptake through increased counter-regulatory hormones, release of cytokines and lipids, and also through direct hepatocyte injury. In addition, drugs often used in COVID-19 treatment, such as corticosteroids, also result in metabolic dysregulation and impaired glucose homeostasis.

New-onset diabetes after SARS-CoV-2 infection or vaccination has been reported. Autoantibody-negative, insulin-dependent diabetes was reported following infection (7). Tang et al. also documented a case of FT1D after receiving the first dose of an inactivated COVID-19 vaccine (5). Individuals with HLA genotypes predisposed to T1DM were diagnosed with FT1D several days after COVID-19 mRNA vaccination (8, 9). Typical symptoms of hyperglycemia and ketoacidosis following vaccination were reported in patients who had also been treated with immune checkpoint inhibitors (10, 11).

Studies have revealed that receptors involved in SARS-CoV-2 viral entry, including angiotensin-converting enzyme 2 (ACE2), neurophilin-1 (NRP1), and transmembrane serine proteases 2 (TMPRSS), were detected in human islet β cells, though at low levels. This may indicate that human islet cells are permissive to SARS-CoV-2 infection. Additionally, clinical data demonstrated that SARS-CoV-2-triggered necroptosis and apoptosis of islets cells were linked to increased glucose levels, a significant viral load, and strong ACE2 expression in β cells (12). Preclinical studies also supported the role of ACE2 in β cell homeostasis: deletion of ACE2 impairs β cell proliferation, decreases β cell mass, and induces β cell oxidative stress and thus decreases insulin secretion. Besides, in high-fat diet mice, de-differentiation of β cells was characterized by a reduction of ACE2 (13). Moreover, molecular mimicry between the SARS-CoV-2 spike protein and human endocrine cells, including pancreatic β cells, has been proposed as a possible element in pathogenesis (14). A recent study using autopsy samples also concluded that the SARS-CoV-2 viral antigen was detected in both endocrine and non-endocrine human pancreas cells, and that the expression of multiple chemokines as well as cytokines were higher in SARS-CoV-2-infected human islets (15). It has also been reported that pancreatic β cells presented with a lower expression of insulin but with a higher expression of glucagon and trypsin1, suggesting that cellular transdifferentiation takes place upon SARS-CoV-2 infection (15). The virus-induced inflammatory cytokines storm, a prothrombotic state, and endothelial derangement *via* ACE2 receptors might also injure β cells function, potentially by affecting the islet microvascular system. Local islet inflammation and systematic oxidative stress after infection might also induce post‐translational protein modifications, enhance the generation of neoepitopes, and thus initiate islet autoimmunity (16).

However, the genotype of classical human leukocyte antigen (HLA) alleles was not determined in this case. A direct causal relationship could not be proven in this patient; however, we regard the SARS-CoV-2 infection as a suspicious trigger of FT1D onset in the gestational setting. More studies on the interactions between SARS-CoV-2 and pancreatic cells are warranted.

In conclusion, we presented a case report of a woman who developed FT1D after SARS-CoV-2 infection during late pregnancy. This is a rare and life-threatening situation, with high stillbirth or miscarriage rates. Clinicians should be aware of the possibility of the onset of FT1D within the COVID-19 background, especially in expectant mothers.

## Data availability statement

The raw data supporting the conclusions of this article will be made available by the authors, without undue reservation.

## Ethics statement

Ethical review and approval was not required for the study on human participants in accordance with the local legislation and institutional requirements. Written informed consent was obtained from the patient for the publication of this case report.

## Author contributions

All authors participated in treating this patient on the ward and were involved in the paper’s conception and analysis as well as interpretation of the lab results. LZ collected and summarized the case data and wrote the first draft of the manuscript; HQ visualized the timeline; and all authors edited, reviewed, and approved the final version of the manuscript. LS is the guarantor of this work and thus has full access to all the data in the study and takes responsibility for its integrity. All authors contributed to the article and approved the submitted version.
